# Masked Face Recognition Using Histogram-Based Recurrent Neural Network

**DOI:** 10.3390/jimaging9020038

**Published:** 2023-02-08

**Authors:** Wei-Jie Lucas Chong, Siew-Chin Chong, Thian-Song Ong

**Affiliations:** Faculty of Information Science &Technology, Multimedia University, Melaka 75450, Malaysia

**Keywords:** masked face recognition, neural network, histogram of gradients, deep learning, recurrent

## Abstract

Masked face recognition (MFR) is an interesting topic in which researchers have tried to find a better solution to improve and enhance performance. Recently, COVID-19 caused most of the recognition system fails to recognize facial images since the current face recognition cannot accurately capture or detect masked face images. This paper introduces the proposed method known as histogram-based recurrent neural network (HRNN) MFR to solve the undetected masked face problem. The proposed method includes the feature descriptor of histograms of oriented gradients (HOG) as the feature extraction process and recurrent neural network (RNN) as the deep learning process. We have proven that the combination of both approaches works well and achieves a high true acceptance rate (TAR) of 99 percent. In addition, the proposed method is designed to overcome the underfitting problem and reduce computational burdens with large-scale dataset training. The experiments were conducted on two benchmark datasets which are RMFD (Real-World Masked Face Dataset) and Labeled Face in the Wild Simulated Masked Face Dataset (LFW-SMFD) to vindicate the viability of the proposed HRNN method.

## 1. Introduction

Masked face recognition (MFR) is a challenging problem to be solved nowadays. With the presence of COVID-19, many people have started to put on their masks to prevent inhaling the virus in the air, and these actions save many people’s life by reducing the transmission rate of COVID-19. However, using MFR led to the challenges and failure of user authentication and verification systems such as the face recognition system. A conventional face recognition system cannot verify or recognize a person’s face that is half-covered by a face mask. This might be a critical problem in security for access control for a person’s authorization in entering restricted areas, labs, or rooms. It will become worse when it comes to an unconstraint public environment. In public areas, criminals can move “undetectably” by wearing a mask since the CCTV fails to capture a criminal’s face when it is covered with a mask. This will further increase the failure rate of a face recognition system when detecting or recognizing masked facial images. In order to overcome exploitation, an MFR system must be designed for face detection and recognition. Overall, there are two consequences when face recognition fails to recognize masked facial images:

1. Crime rate will increase since criminals can avoid camera face recognition systems when they put on the mask.
2. Face recognition-based biometric systems will have a low true acceptance rate (TAR) for access control or identification purposes.

According to Déniz’s research on face recognition using histograms of oriented gradients (HOG) [1], the authors proposed three essential steps for using the HOG to build a face recognition system. Firstly, the authors implemented the HOG descriptor for grid extraction. Secondly, they fused the HOG descriptor with different scales. Third, they performed a dimension reduction process to reduce noise for preventing overfitting. This work inspires us to provide a solution for building face recognition that can recognize mask facial images with the deep learning approach with the combination of HOG descriptors. 

This paper will discuss the proposed method, Histogram-based Recurrent Neural Network (HRNN) MFR, which can recognize masked facial images and increase the face recognition system’s TAR. TAR, which is also called true match rate (TMR), is a metric evaluation for biometrics authentication using a probability formulation by accepting an authorized person. The probability in TAR in this experiment is presented as a percentage of how many times a label’s masked facial images have been matched correctly with the unmasked images. 

HRNN is a combination based on feature extraction of the histogram of oriented gradient (HOG) and deep learning approach recurrent neural network (RNN). The combination of these two approaches works well and could achieve a high TAR based on evaluating two benchmark datasets. In this context, the HOG feature descriptor extracts the gradient and orientation from the normalized dataset in the feature extraction process. This process helps to minimize the feature size by extracting the essential element from the data for further training processes and also reduces the computational burden of the machine. Next, the deep learning process with the RNN trains the feature extracted from the HOG. The reason for choosing the RNN deep learning approach is that each output of the RNN is dependent on the previous output. The backpropagation mechanism in RNN uses the actual output and puts it back into the neurons in the neural network with some mathematical calculation to give the desired result. 

After the training process, the model is evaluated with different categories on each benchmark dataset. The detailed experiment will be presented in Section 5 of the paper. The hyperparameter tuning process helps to reduce the problem encountered during the deep learning process, including the overfitting and underfitting problem. The experiment result will be evaluated with TAR, recorded, and compared to each category.

Section 2 of the paper will discuss the related research work and literature review of face recognition and MFR. Section 3 shows the motivation and contribution of this paper and the inspiration for the proposed method. Section 4 of the paper will explain the proposed solution with the implementation of the algorithm in the proposed method. Section 5 will evaluate the proposed method’s performance and experimental analysis with state-of-the-art methods. The last section of this paper concludes the works.

## 2. Literature Review

This part of the paper discusses the technologies used in face recognition and MFR, including feature extraction, deep learning methods, and classification algorithm in machine learning. Moreover, some existing face recognition and MFR approaches are reviewed and analyzed to understand the implementation and usage of each method in the research. This section aims to identify the research gaps and understand the current situation of technologies used in this machine learning field. Therefore, the finding will be essential to produce a reliable MFR system.

### 2.1. Feature Extraction Approaches

The feature extraction acts as a machine learning catalyst to accelerate the processes and perform vector extraction from the original data for further purposes. The MFR system’s performance can be improved during the module’s training and testing phases with a good feature extraction procedure. Some good feature extraction techniques are widely used in machine learning, and it is worth further understanding in detail. Those feature extractions are principle component analysis (PCA) [2], HOG [1], linear discriminant analysis (LDA) [3], and transfer learning feature extractors such as VGG16 [4].

PCA is a data-simplified technique that helps to reduce the large data size by extracting the feature from the original data. The main objective of this approach is to reduce the dimensionality of multivariate data whilst preserving as much of the relevant information as possible [5]. It is categorized as unsupervised learning that depends on the data input without any actual data sample. The algorithm in PCA performs a linear transformation in which the input data are transformed into a new coordination example: principle components, linear functions, variances, and a new set of variables [5]. 

On the other hand, HOG is a feature extraction approach often used to extract the feature from image data. The HOG algorithm’s focal point is the image data’s shape. It detects the edge by providing the magnitude direction of the edges from the data in gradient and orientation feature representation. In addition to that, the gradient and orientation extraction are calculated in localized portions [6]. This means the whole image data are separated into a minor part of the region, and the calculation occurs in each region. Finally, a histogram from the separated region using the pixel value is constructed for classification [6]. 

LDA is a pre-processing algorithm used in machine learning before the classification step. It is a dimension reduction technique that projects the feature of a higher dimension onto a lower dimension to prevent some errors from occurring in large feature vectors and minimize the dimensional cost and resources. Originally this algorithm was designed for solving binary classification problems. The multi-classification method was introduced with a multiple discriminant analysis (MDA) [7]. LDA was widely used to solve image recognition problems in machine learning models [8].

The transfer learning method can be used as a feature extractor in machine learning. Some examples of transfer learning models include VGG16, ResNet, Xception, and Inception. These approaches are flexible by using a pre-trained model for feature extraction and image pre-processing for integrating into another different model. The VGG family includes VGG16 and VGG19, while GoogLeNet categories include Inception and the Residual Network ResNet50, ResNet34, and ResNet18.

### 2.2. Deep Learning Approaches

Deep learning is a part of machine learning and is widely used in computer vision, such as image classification, image recognition, text recognition, pattern recognition, and cryptocurrency predictions. There are many practical deep learning algorithms which are convolutional neural networks (CNN) [9], recurrent neural networks (RNN) [10], self-organizing maps (SOM) [11], and generative adversarial networks (GANs) [12]. These deep learning algorithms are classically used in computer vision, text recognition, currency prediction, and pattern recognition. 

CNN or ConvNet is a deep learning algorithm that is famous in image classification. The algorithm uses an input image and assigns weights to the object in the data, which are well labeled by categories. With the data augmentation in CNN, the size of the training set could be increased to prevent overfitting problems when using large-size datasets [13].

RNN is categorized as an artificial neural network (ANN). This approach is widely implemented in solving time series and sequential data problems. Many problems can be solved with RNN, such as language translation, speech recognition, and natural language processing. They are distinguished by their “memory” as they take information from prior inputs to influence the current input.

In the paper by [14], the authors proposed a face recognition system (AdaFace) with new loss functions which overcome the difficulties of recognizing low-quality images. The objective can be achieved with an adaptive margin function which enhances the image quality with the feature norms. With the proposed method AdaFace loss function, the result achieves 99.83 TAR in the high-quality category, 97.39 TAR in IJB-C (mixed quality), and 76.11 TAR in the low-quality image category. There are some limitations and weaknesses in this proposed method. The AdaFace is weak in handling noisy data in the large-scale dataset and cannot deal with a mislabeled sample problem [14].

### 2.3. Classification Approaches

The classification algorithm is a supervised learning technique used to identify a new observation’s labels on the training data [15]. For binary classification, the task was classifying cat and dog images by giving an input image for prediction. It predicts a model when given the input data, such as labeled data, categorized data, and targets. Many practical classification algorithms for image classification are widely implemented in machine learning. Each approach will be explained and discussed in this section. 

The support vector machine (SVM) [16] is a universal classification algorithm for solving an image classification problem. It is categorized as supervised machine learning and can produce a good result in binary classification. SVM can perform exceptionally well for well-labeled datasets in the training phase. 

The decision tree [17] is a machine-learning model that builds like a tree structure. This approach is categorized as supervised learning in machine learning. The way this classifier works is to divide data into smaller subsets in the decision tree by using the mathematics rules (if-then). This algorithm is often used in image classification, prediction, regression, and data analytics tasks. 

K-nearest neighbors (KNN) [18] is categorized as a pattern recognition algorithm in machine learning. It kept and saved its learnings from the training data by analyzing the data among data in n-dimension space. In addition, this algorithm targets finding the invisible data, k, the nearest related data point in upcoming predictions. This method is suitable for predicting unconstrained datasets. 

Naïve Bayes [19] is a machine learning classifier; it is classified as a probabilistic algorithm, and the algorithm of this method is based on Bayes’ Theorem. The algorithm calculates the probability of the input data being categorized into one or more groups of categories or not. This approach is widely used in classifying text analysis, such as categorizing comments, emails, and news articles into topics, subjects, or tags to further organize the data into future predictions.

### 2.4. Existing Face Recognition Methods and Performance

Ejaz [20] presented an MFR approach using the multi-task cascaded convolutional neural network (MTCNN) method, whereby GoogleFaceNet is used as transfer learning feature extraction and the SVM classifier is used as the classification method for the proposed method. However, the author stated that the disadvantage is that the proposed method will not perform well in recognizing several masked types, such as the color and model of the mask. The proposed method can be enhanced by embedding more work to overcome weaknesses. 

In Anwar [21], the authors utilized an open-source masks simulation tool, MaskTheFace, to generate a masked facial dataset for experimental purposes. In the experiment, the author implemented a pre-trained model, FaceNet and the results show that the model can improve 38% of true positive rate (TPR). Three masked face simulated datasets were tested: LFW-SM (combined), VGG Face2-mini, one real-world dataset, MFR2, and VGGFace2-mini-SM 1. In the experiment of LFW-SM dataset evaluation, the result achieves a 97.25% accuracy which is the best performance among the others. 

Hang [22] proposed an MFR method to solve the NIR-VIS training and testing method problem. The authors adopted a novel heterogeneous training approach that maximizes the share information. The domain-invariant face representation approach in the proposed method can perfectly cover the masked region on the facial image. The authors used 3D reconstruction to integrate the masked face data to ensure the masked face dataset is sufficient for training and testing. The results show the highest accuracy of 98.60% with HSST (Triplet) method in CASIA NIR-VIS 2.0 dataset as compared with the other two datasets, which are Oulu-CASIA NIR-VIR (91.3%) and BUAA-VisNir (98.40%). 

Desai [23] adopted an object detection algorithm, You Only Look Once (YOLO), to propose MFR in the smartphone security system. The job of the YOLO algorithm is to recognize masked and unmasked facial images based on their unique labels. The authors created their datasets consisting of the facial images of six persons to test the proposed method. The authors conducted several experiments to test the performance of different variations of the YOLO algorithm: YOLOv3 TINY, YOLOv4 TINY, YOLOv3, and YOLOv4, and the results are presented in a table for comparison purposes. YOLOv4 has the best performance among the other variation, which is able to achieve an 84% recognition rate. 

Fatema [24] used a transfer learning model to retrain the FaceNet with Residual Neural Network variation (ResNetv1 and ResNet50 architecture) in MFR as the proposed method. The author highlighted the challenges of the validation set during the experiment. They breakthrough successfully by using the hyperparameter tuning method to stabilize the experiment. The authors finalized the experiment by implementing the K-Nearest Neighbor (KNN) as the classification output. 

Wu [25] proposed an MFR approach for Contactless Distribution Cabinet. Due to the COVID-19 pandemic, the authors designed an MFR system in a contactless cabinet that allows people to collect their couriers without direct contact with another person, reducing virus transmission. In the proposed method, the authors use the local constrained dictionary learning algorithm (LCDL) to detect and extract face images from the dataset. In order to decrease the resolution reduction during the subsampling process, the authors selected the dilated convolutional method as the solution. They implemented an attention mechanism to stabilize the training process to produce better training output. Additionally, the convolutional neural network was added to enhance the recognition rate of the system.

In Deng’s MFR InsightFace track report [26], a research analysis is conducted using InsightFace. This research tests determining a face recognition system’s performance on different facial images such as masked, children, and multi-racial images. The authors construct an online face recognition model by two training datasets, MS1M [27] and Glint360K [28]. A large-scale dataset containing 7000 identities for the test set, 14,000 for the children face test set, and 242,000 for the multi-racial test set by manual collection. 

In the paper by Deng [29], the authors proposed an Additive Angular Margin Loss (ArcFace) for extracting highly discriminative features in a face recognition system. Due to its perfect coordination to geodesic distance on a hypersphere, the proposed method has a precise geometric interpretation. The primary goal of this research is to stabilize the training process and improve the discrimination capability of the face recognition model. This experiment was conducted using a variety of large-scale datasets, including LFW, CASIA, VGGFace2, YTF, and others. 

MFCosface [30] is an MFR approach based on large-margin cosine loss. The authors created a simulated masked face dataset through MTCNN and covered the facial region’s lower part with a mask template. Because the dataset is unsuitable for the triple loss function, the authors used large-margin cosine loss for training. It can map all the feature samples in feature space with smaller intra-class and larger inter-class distances. To further increase the accuracy, the combination of the convolutional block attention and Inception-Resnet are integrated into the proposed method to raise the weight of the exposure facial region in the feature map. The experiment is evaluated with several different datasets: CASIA-FaceV5_m [31], VGGFace2_m [32], RMFD [33], MFR2 [34], and LFW_m [35].

## 3. Motivation and Contribution

This section discusses the motivation and contribution of the proposed method in MFR. In recent years, wearing face masks has become a habit among people to avoid spreading COVID-19 viruses. However, this trend eventually complicates the face recognition system’s failure to recognize masked facial images and causes the system to have a low TAR. 

The main problem that causes the system to fail to recognize masked facial images is that the existing facial recognition algorithms cannot accurately detect a human’s facial region. Once the system fails to detect the facial region, the system malfunctions in extracting the feature from the images. On the other point, leaks of information gather because half of the facial region is covered with “unknown things” from the face recognition perspective. To overcome the weaknesses of the face recognition system, an MFR system is necessary for enhancing the existing facial recognition system, which allows the system to recognize and authorize a person effectively in the mask-on condition. 

In this paper, we proposed an MFR system using an integration of feature extraction and a deep learning approach named HRNN. The main idea of the proposed method is to utilize the HOG to perform feature extraction from masked facial images and the RNN for the deep learning processes. The proposed method can enhance the training and testing speed compared to a default CNN. In addition, the HOG can perform better than PCA during the training phase. 

The main challenge of this experiment Is to build a reliable face recognition system that can recognize masked facial images MFR and a system that can hit a high recognition rate for the testing phase. First, two open-source datasets are selected, RMFD and LFW-SMFD, to test the model. The datasets are fetched to the pre-processing data phase. In this phase, the normalization and feature extraction processes are performed. Next, the extracted feature vector is fed to RNN for deep learning processes. 

Overall, the main contributions of the proposed method are:

- Address the weaknesses of the conventional face recognition system. The proposed method can effectively recognize unconstrained masked face images by using a deep learning approach through two benchmarked datasets;
- Solve overfitting and underfitting problems by using hyperparameter tuning and HOG feature extraction. The proposed method can fit large, scaled datasets without having overfitting and underfitting problem, which will be substantiated in the experimental section;
- Solve slow training and testing speed while using big datasets. The proposed method improves the training and testing speed performance by using a large-scale dataset compared with the default CNN settings.

The work was inspired by the benchmarked MFR framework using MTCNN [20] designed with FaceNet feature extraction and SVM adopted classifier to predict the output of the experiment. Compared with our proposed HRNN method, we use a non-pre-trained feature extraction method, the HOG feature descriptor. This allows the MFR system to speed up the feature extraction phases. Next, the RNN is implemented to train and predict the feature. HRNN adopts a lighter architecture than the benchmarked framework during the feature extraction and classification phase, in which the computational time, training, and testing speed are enhanced.

Furthermore, compared with the benchmarked framework using the cropping approaches to ensure the system can extract features more, focusing on facial region, our proposed method only uses greyscaling during the feature extraction process. This is because the data are already being processed with cropping. Therefore, there is no necessity to repeat the process. Lastly, the RNN can solve overfitting and underfitting problems using the hyperparameter tuning method, which helps with performance accuracy.

## 4. Proposed Solution

This section will discuss the overall algorithm and experimental processes. Through the reading of the paper [36], we understand that the benchmarked framework uses the FaceNet and SVM feature extraction and classification, respectively. This motivates us to propose a more time-saving method that uses the HOG rather than FaceNet for feature extraction and RNN as the deep-learning phase. Our proposed method has the same ability in recognizing various types of masked and different categories of labels. It can solve the low acceptance rates of conventional facial recognition systems and speed up the training time for large-scale datasets. It combines feature extraction and the deep learning mechanism to build a reliable MFR system.

**Figure 1:**
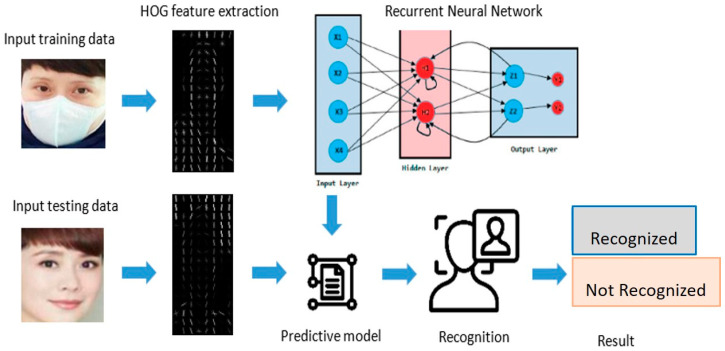
Overall process of the proposed method.

Figure 1 shows an overall diagram of the proposed HRNN MFR using an RNN with a HOG feature extraction. All images, including masked and unmasked facial images, are imported from the dataset. The size of all input images is resized from 160 × 160 pixels to 28 × 28 pixels by Equation (1) to fit the neural network. Equation (1) shows the process of resizing an image. Equation (1), x represents the width pixel of the image, *y* represents the height pixel of the image, and $R_{img}\;$ is the resized image. Before the feature extraction process, all images are normalized with the greyscaled process Equation (2). Equation (2) shows the greyscale equation for the normalization process. RGB is an additive color in computer vision. It refers to the red, green, and blue pixels of a display. In Equation (2), x, y, and z are constant values that multiply with R, G, and B to build $G_{img}\;$ a greyscaled image.

(1) $$R_{img}=resize\left(x,y\right)$$

(2) $$G_{img}=R_{img}\left(xR+yG+zB\right)$$

Next is the feature extraction process, all the $G_{img}$ are fetched to Equation (3), the HOG feature extraction process, to extract the feature vector for each image. Equation (3) shows the HOG feature extraction process. The hog in Equation (3) is built with the gradient magnitude M Equation (4) and orientation θ Equation (5). In Equation (4), x represents the total width pixel of the image, y represents the total height pixel of the image, M is the total gradient/magnitude. In Equation (5), θ represents the orientation/direction.

(3) $$\mathrm{HO}G_{img}=hog(G_{img})$$

(4) $$M=\sqrt{(x^{2}+y^{2}})$$

(5) $$\tan\left(\theta \right)=\frac{y}{x}\;\theta =\mathrm{atan}\left(\frac{y}{x}\right)$$

Furthermore, a min–max normalization process is implemented to reduce the training data’s total feature size. Equation (6) refers to the process of min–max normalization $N_{minmax}$. The main reason for this process is to transform all the features into 0 and 1 data. This helps to speed up the process when we train the data.

(6) $$N_{minmax}=\frac{S\left(\mathrm{HO}G_{img}\right)-MIN\left(rgb\right)}{MAX\left(rgb\right)-MIN\left(rgb\right)}(new\_\;MAX\left(rgb\right)-new\_MIN\left(rgb\right)+new\_\;MIN(rgb)$$

In Equation (6), $S\left(\mathrm{HO}G_{img}\right)$ represents the size of a HOG feature vector in pixels, MIN(rgb) refers to the minimum value of the RGB, MAX(rgb) represents the maximum value of RGB. new_MIN(rgb) represent the new size of MIN(rgb) and new_MAX(rgb) represent the new size of MAX(rgb). Next, let $N_{minmax}=N$, N represent features normalized by Equation (6), and N is trained by using RNN. Equation (7) shows the equation of RNN.

(7) $$h^{\left(t\right)}=f\left(h^{\left(t-1\right)},x^{\left(t\right)};\theta \right)$$

In Equation (7), $h^{\left(t\right)}$ represents the current hidden state in the neural network, the function of f and its past hidden state of $h^{\left(t-1\right)}$. $x^{\left(t\right)}$ refers to the current input data and θ refers to the parameter input of function f. The data were trained with the sequential model settings in RNN with the LSTM architecture Equation (8) and combined with the Dropout Equation (11) and Flatten Functions Equation (12) as the parameter. At last, the neural network is optimized with the Adam function Equation (15), sparse categorical cross-entropy as the loss function Equation (16) and Softmax Equation (17) as the activation function.

(8) $$\begin{array}{l} f_{t}=\sigma_{g}\left(W_{f}\times x_{t}+U_{f}\times h_{t-1}+b_{f}\right)\\ i_{t}=\sigma_{g}\left(W_{i}\times x_{t}+U_{i}\times h_{t-1}+b_{i}\right)\\ o_{t}=\sigma_{g}\left(W_{o}\times x_{t}+U_{o}\times h_{t-1}+b_{o}\right)\\ {c^{\prime }}_{t}=\sigma_{c}\left(W_{c}\times x_{t}+U_{c}\times h_{t-1}+b_{c}\right)\\ c_{t}=f_{t}\cdot c_{t-1}+i_{t}\cdot {c^{\prime }}_{t}\\ h_{t}=o_{t}\cdot \sigma_{c}\left(c_{t}\right) \end{array}$$

In Equation (8), $f_{t}$ represents the forget gate, $i_{t}$ represents the input gate, $o_{t}$ represents the output gate, $c_{t}$ refers to the cell state, $h_{t}$ refers to the current hidden state and $h_{t-1}$ refers to the previous hidden state. $\sigma_{g}$ and $\sigma_{c}$ represent sigmoid Equation (9) and tanh Equation (10) activation functions, respectively. $W_{f}$, $W_{o}$, $W_{i}$, $W_{c}$, $U_{f}$, $U_{i}$, $U_{o}$ and $U_{c}$ represents the weight metrics. $b_{f}$, $b_{i}$, $b_{o}$ and $b_{c}$ represent the biases.

(9) $$S\left(x\right)=\frac{1}{1+e^{-x}}$$

(10) $$\tanh\left(x\right)=\sinh\left(x\right)\cosh\left(x\right)\;\tanh\left(x\right)=e^{x}-e^{-x}e^{x}+e^{-x}$$

(11) $$O_{i}^{h}=\sigma \left(S_{i}^{h}\right)\;O_{i}^{h}=\sigma \left(\sum_{l<h}\sum_{j}w_{ij}^{hl}O_{j}^{l}\right)$$

(12) $$f=\frac{\left(a-b\right)}{a}$$

In Equation (11), $O_{j}^{l}$ = $I_{j}$ where the $O_{i}^{h}$ output is based on the unit of i in the layer of h. In Equation (12), a represents the semimajor axis and b represents the semiminor axis. Equation (15) shows the process of the Adam optimizer. The Adam optimizer is made up of two essentials component, which are momentum Equation (13) and root mean square propagation (RMSP) Equation (14).

(13) $$w_{t+1}=w_{t}-\alpha m_{t}\;m_{t}=\beta m_{t-1}+\left(1-\beta \right)\left[\frac{\delta L}{\delta w_{t}}\right]$$

(14) $$w_{t+1}=w_{t}-\frac{\alpha_{t}}{{\left(V_{t}+\varepsilon \right)}^{\frac{1}{2}}}*\left[\frac{\delta L}{\delta w_{t}}\right]\;V_{t}=\beta V_{t-1}+\left(1-\beta \right)*{\left[\frac{\delta L}{\delta w_{t}}\right]}^{2}$$

In Equation (13), $m_{t}$ refers to the aggregate of gradients at time t, $m_{t-1}$ refers to the aggregate of gradients at a previous time. $w_{t}$ represents the weights at current t.$\;w_{t+1}$ represents the weights at future t. α refers to the learning rate. δL refers to the derivative of the loss function, $\delta w_{t}$ represents the derivative of weight in t. β represents the moving average parameter. Equation (14), $V_{t}$ represents the sum of the square of past gradients. ε refers to a small positive constant. Equation (15) shows the dropout process in the neural network. Equation (16) shows the sparse categorical cross-entropy as the loss function in the neural network.

(15) $$w_{t+1}=w_{t}-\hat{m_{t}}\left(\frac{\alpha }{\sqrt{\hat{V_{t}}}+\varepsilon }\right)$$

(16) $$L_{CE}=-\sum_{i=1}^{n}t_{i}\log\left(p_{i}\right)$$

where n is the number of classes, $t_{i}$ is the truth label and $p_{i}$ represents the Softmax probability in $i^{th}$ class.

(17) $$\sigma ({\vec{z}}_{i})=\frac{e^{z_{i}}}{\sum_{j=1}^{K}e^{z_{j}}}$$

Equation (17) shows the process of the Softmax activation function. σ refers to the Softmax, ${\vec{z}}_{i}$ represents the input vector, $e^{z_{i}}$ represents the standard exponential for the input vector, K refers to the number of classes in the multiclassifier.$\;e^{z_{j}}$ represents the standard exponential for the output vector.

Algorithm 1 shows the overall process of the proposed HRNN with the expected inputs and outputs.
**Algorithm 1.** Histogram-based Recurrent Neural Network**Input:**d = Masked and unmasked facial images imported from datasets. $d_{max}$ represents the maximum number of labels in the dataset. **Output:** Prediction Model. 
  1.Let c=1, c represents a counter for the amount of loop.  2.$d_{max}$ = the maximum number of labels in the dataset.  3.Load d into the experiment.   4.**repeat**  5.  Compute d with Equation (1) for $R_{img}$.  6.  Compute $R_{img}$ with Equation (2) for $G_{img}$.  7.  Compute $G_{img}$ with Equation (3) for $\mathrm{HO}G_{img}$.  8.  c=c+1  9.**until**$c==d_{max}$  10.Compute $\mathrm{HO}G_{img}$ with Equation (6) for $N_{minmax}$.  11.Compute $N_{minmax}$ with Equation (7) for $h^{\left(t\right)}$.  12.**return** Prediction Model.

## 5. Experimental Results

This section will evaluate the performance and results of the HRNN. Two benchmarked datasets: Labeled Face in the Wild Stimulated Masked Face Dataset (LFW-SMFD) [35] and Real-world Masked Face Dataset (RMFD) [33] are tested with the proposed method. There are 243 total labels and 1996 facial images in the RMFD dataset. Additionally, LFW-SMFD has 2271 total labels and 5442 samples of facial images. The Testdir (TD) is a self-build dataset to test and predict the possibilities of the methods, models, and algorithms that work for the actual benchmark datasets. The TD dataset is a mini dataset with a subset of both benchmarked datasets from RMFD and LFW-SMFD; it consists of 36 total labels and 251 facial images. The primary purpose of creating the TD dataset is to predict the performance of different approaches used in the experiment in a shorter time. The result shows that the TD dataset could test a single cycle of the experiment within 10 min rather than using the whole benchmarked dataset with a longer time for a single experiment. Table 1, Table 2 and Table 3 present the experimental results from three different datasets.

Table 1 shows the experiment results in the TD dataset. The main objective of the test is to find the effectiveness of each method that suits the benchmarked datasets and shorten the experimental processing time. All of the epochs in the experiment are fixed at 50 to test the approaches in each experiment, which prevents the experiment from having inequity results. At first, experiments No. 101 and 102 are tested with the KNN approaches. No. 101 has no feature extraction method implemented, and No. 102 has the ResNet50 as the feature extraction approaches. The results show that No. 101 have 3% higher than No. 102, which is 33.28% and 30.80%. Next, experiments No. 109, 110, 111, and 112 are tested with the SVM approaches. Each experiment is implemented using different feature extraction types: ResNet50, VGG16, Inceptionv3, and EfficientNetB7. The results of all four categories are unsatisfactory and have low TAR in the experiment. Experiment No. 109 has the lowest TAR, which is 7.22% and No. 111 achieves 42.45% of TAR. All of the remains experiments are tested on the RNN method. The RNN method fits well with the TD dataset. Experiment No. 108 tests the combination of RNN and SVM approaches. It shows a bad result of 2.4% TAR, and it can be concluded that this method is unsuitable for the dataset. Experiment No. 103 is tested with the RNN with no feature extraction method, the result shows a high TAR of 99.19%, and it might work for the benchmarked datasets. To find the answer, the feature extraction of the HOG approach is added to RNN, which is experiment No. 113. The result shows that it perfectly fits well to the datasets and achieves a 100% TAR. Before this category of experiment ends, the HOG feature extraction is replaced with another transfer learning module as a feature extraction same as the SVM experiment test. However, the results show that the experiment results have low TAR. Experiment No. 114 implements a PCA dimension reduction process for the RNN and HOG approach. It achieves a 30.40% TAR. With the result of Table 1, we can conclude that the RNN with the feature extraction approach can outperform other approaches and give reliable results.

Table 2 shows the comparison result for the RMFD benchmark dataset. All of the epochs in the experiment are set at 50. Experiments No. 201 and 202 are tested with the KNN approach. No. 201 has no feature extraction and No. 202 have the feature extraction with the ResNet50 transfer learning approach No. 202 has higher TAR than No. 102, which is 34.88% and 29.59%, respectively. Experiment No. 203, 204, 205, and 206 on the RNN approach. At first, No. 203 is conducted to test the performance of RNN in RMFD dataset with no feature extraction implemented. The result of No. 203 seems good and achieves a TAR of 91.63%. Experiment No. 204 tested the transfer learning feature extraction approach. In the table, there is only one transfer learning module that is tested. This is because, according to Table 1, another transfer learning module shows that it performs terribly with low TAR.

Nevertheless, the Inceptionv3 approach has always had the highest TAR among the other transfer learning modules. The experiment on the transfer learning approach Inceptionv3, which is No. 204, can achieve a 48.42% TAR. Experiment No. 205 uses the approach from Table 1 which has the highest TAR compared with other experiments, which perform a 99.60% TAR in RMFD benchmarked dataset. At last, an extra experiment, No. 206, is tested to find out what happens when the epoch goes on after 50. The experiment has 100 epochs and achieves a 98.67%, which is 1% lower than No. 205. Therefore, we can conclude that the combination of the RNN and HOG method works well in the RMFD dataset and the larger epoch will not necessarily increase the TAR.

Table 3 shows the experiment result of the LFW-SMFD benchmark dataset. All of the epochs in the experiment are fixed at 50. The first experiment tested with the LFW-SMFD dataset is experiment No. 301. The experiment was tested with the KNN approach, and no feature extraction was added. The results show that No. 301 can achieve only 18.53% TAR. Next, the transfer learning approach ResNet50 as feature extraction was tested with the KNN, which is No. 302 and achieves a 43.70% TAR. Experiment No. 303 tested with the VGG16 as the feature extraction approach with KNN. Experiment No. 303 achieves a 42.91% TAR. The remaining experiment is conducted using the RNN with different feature extractions to observe the difference in the performance. Experiment No. 304, using RNN with no feature extraction method, can perform a TAR of 47.35%. Next, No. 305 uses an Inceptionv3 as the feature extraction method and achieves an 11.93% TAR. At last, experiment No. 306 which is the best approach in Table 1 and Table RNN with HOG feature extraction, performs well in the LFW-SMFD dataset and achieves the highest TAR of 99.56%. We can conclude that the RNN with HOG approach is the best method for both benchmark datasets.

**Figure 2:**
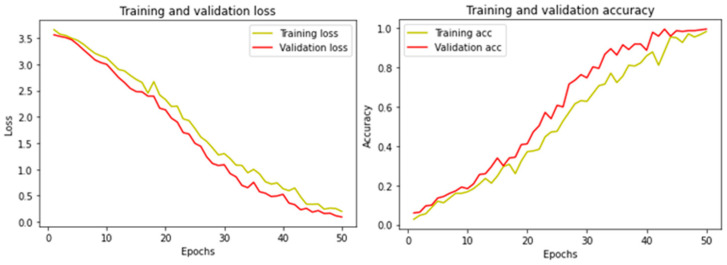
Training performance with proposed method in RMFD dataset.

Figure 2 shows the RMFD evaluation graph for training and validation loss (left) and training and validation accuracy (right) with the proposed method. The loss graph refers to how bad or good the model performs after each epoch while training. The loss graph in Figure 2 started from above a 3.5 loss in the 0th epoch and continuously dropped until a 0.3 loss in the 50th epoch, representing good training for the model. On the right site, the accuracy graph refers to evaluating the model performance in an explicable way. The accuracy graph in Figure 2 started from 0 accuracies in the 0th epoch until 0.99 accuracies in the 50th epoch in which the model is well trained.

**Figure 3:**
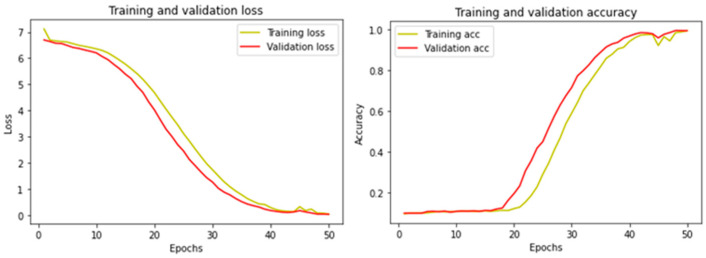
Training performance with proposed method in LFW-SMFD dataset.

Figure 3 shows the LFW-SMFD evaluation graph for training and validation loss (left) and training and validation accuracy (right) with the proposed method. The loss graph in Figure 3 started from above a 6.8 loss in the 0th epoch and continuous dropping until 0.1 loss in the 50th epoch. On the right site, the accuracy graph in Figure 3 started from 0 accuracies in the 0th epoch until 0.99 accuracies in the 50th epoch.

Table 4 shows the computational performance comparison on both benchmark datasets. The experiment is conducted on the processor Intel(R) Core(TM) i7-6700HQ CPU @2.60GHz 2.59 GHz, 16.0 GB RAM, 64-bit operating system, x64-based processor, NVIDIA GeForce GTX 1060. 

According to Table 4, the HRNN method needs only 20 min of training time compared to the RNN, which requires 46 min of training time in the RMFD dataset. Next, for the LFW-SMFD dataset, the HRNN has also faster than RNN, which is 76 min and 182 min in training time and 0.0463 img/s and 0.1839 img/s in testing time, respectively. The result shows that HRNN has improved both the benchmark dataset’s training and testing computational time.

Table 5 exhibits the performance comparison of the proposed method and state-of-the-art methods. 

At first, the MFR with ResNet50 achieved a 47.19% accuracy. According to the experiment conducted in Table 1, the ResNet 50 has a poor performance when combined with the RNN. The proposed method changes the feature extraction method to HOG to overcome the problem. Secondly, the MFR using FaceMaskNet-21 reaches 88.92% accuracy. It uses the FaceMaskNet-21 to produce a 128-dimension encoding to support the recognition system. FaceMaskNet-21 is a deep neural network with convolutional layers, ReLU, cross-channel normalization, maxpooling, and Softmax. FaceMaskNet-21 is an exemplary architecture for MFR. To further improve the accuracy, the proposed method implements an extra feature extraction process to further extract the feature from the data. As a result, the proposed method has successfully increased the accuracy by integrating the HOG after a CNN model. Next, mask face recognition using MFCosface achieves 98.54% accuracy. The method uses large-margin cosine loss to build the MFR system. In the experiment, the proposed method has a better computational time with the RNN and HOG combination than the MFCosface method. MFR using MTCNN, SVM, and FaceNet approach results in 98.10% accuracy. It uses the MTCNN for facial detection, FaceNet for feature extraction, and support vector machine (SVM) classification. The proposed method does not implement the facial detection algorithm such as MTCNN since the dataset used for the proposed method is cropped. In addition, we noticed that the FaceNet and SVM do not perform as well as the HOG in the experiment. In addition, another state-of-the-art method of the LCDL approach, which is a variant of the LCD, could achieve a slightly lower accuracy of 98.00% compared with the proposed method. In summary, the proposed method uses the HOG feature descriptor as the feature extractor, and RNN as the deep learning outperforms other state-of-the-art methods with the highest accuracy of 99.56%.

## 6. Conclusions

In conclusion, a reliable MFR system can strengthen the security or access control of restricted areas and lower the intrusion from unauthorized persons. In the experiment, we can conclude that the proposed method, HRNN, will be able to recognize masked facial images and has a fast computational speed with an extensive training dataset. The proposed method can achieve 99% of TAR by evaluating both benchmark datasets: RMFD and LFW-SMFD. Moreover, according to the result in Section 5, HRNN can solve the overfitting problems by tuning the hyperparameter of the neural network. However, there is also some limitation that we discover during the experiment. The data loading process is highly time-consuming before the pre-processing image phase. The larger the dataset used, the more time is needed for the data loading process. Overall, the combination of feature extraction based on the HOG and RNN works well with a high TAR rate. In the future, we will grind ourselves to overcome the limitation of data loading issues. In addition, it is necessary to test the proposed method with different masked face datasets and use another different approach to enhance the MFR system.

## Figures and Tables

**Table 1:** Comparison of different approaches and results in TD dataset.

Experiment (No.)	Method	Dataset	Feature Extraction	Epoch	TAR (%)
101	KNN	TD	None	50	33.28
102	KNN	TD	ResNet50	50	30.80
103	RNN	TD	None	50	99.19
104	RNN	TD	ResNet50	50	10.40
105	RNN	TD	VGG16	50	4.01
106	RNN	TD	Incecptionv3	50	60.26
107	RNN	TD	EfficientNetB7	50	5.91
108	RNNSVM	TD	None	50	2.40
109	SVM	TD	ResNet50	50	7.22
110	SVM	TD	VGG16	50	12.08
111	SVM	TD	Inceptionv3	50	42.45
112	SVM	TD	EfficientNetB7	50	8.01
113	RNN	TD	HOG	50	100.00
114	RNN	TD	HOG + PCA	50	30.40

**Table 2:** Comparison of different approaches and results in the RMFD dataset.

Experiment (No.)	Method	Dataset	Feature Extraction	Epoch	TAR (%)
201	KNN	RMFD	none	50	34.88
202	KNN	RMFD	ResNet50	50	39.59
203	RNN	RMFD	none	50	91.63
204	RNN	RMFD	Inceptionv3	50	49.42
205	RNN	RMFD	HOG	50	99.60
206	RNN	RMFD	HOG	100	98.67

**Table 3:** Comparison of different approaches and results in the LFW-SMFD dataset.

Experiment (No.)	Method	Dataset	Feature Extraction	Epoch	TAR (%)
301	KNN	LFW-SMFD	None	50	18.53
302	KNN	LFW-SMFD	ResNet50	50	34.70
303	KNN	LFW-SMFD	VGG16	50	42.91
304	RNN	LFW-SMFD	None	50	47.35
305	RNN	LFW-SMFD	Inceptionv3	50	11.93
306	RNN	LFW-SMFD	HOG	50	99.56

**Table 4:** Comparison of computational speed and time in benchmark dataset.

Methods	Datasets	Training (min)	Testing (img/s)
RNN	RMFD	46	0.1133
HRNN	RMFD	20	0.0397
RNN	LFW-SMFD	182	0.1839
HRNN	LFW-SMFD	76	0.0463

**Table 5:** Comparison to other state-of-the-art methods.

Methods	Accuracy (TAR%)
MFR ResNet50 [24]	47.19
MFR FaceMaskNet-21 [37]	88.92
MTCNN+SVM+FaceNet [20]	98.10
MFR LCDL [25]	98.00
MFR MFCosface [30]	99.33
Proposed method, HRNN	99.56

## Data Availability

This section provides details regarding where data supporting report results can be found and the links to publicly archived datasets analyzed or generated during the study. The LFW-SMFD are available at https://www.kaggle.com/datasets/muhammeddalkran/lfw-simulated-masked-face-dataset, accessed on 1 February 2022. RMFD dataset is available at https://github.com/X-zhangyang/Real-World-Masked-Face-Dataset, accessed on 1 February 2022.
